# Bivalent chromatin: a developmental balancing act tipped in cancer

**DOI:** 10.1042/BST20230426

**Published:** 2024-02-22

**Authors:** Eleanor Glancy, Natalie Choy, Melanie A. Eckersley-Maslin

**Affiliations:** 1 Peter MacCallum Cancer Centre, Melbourne, Victoria 3000, Australia; 2 Sir Peter MacCallum Department of Oncology, The University of Melbourne, Melbourne, Victoria 3010, Australia; 3 Department of Anatomy and Physiology, The University of Melbourne, Melbourne, Victoria 3010, Australia

**Keywords:** bivalency, bivalent chromatin, epigenetics, gene expression and regulation, histone methylation, methylation

## Abstract

Bivalent chromatin is defined by the co-occurrence of otherwise opposing H3K4me3 and H3K27me3 modifications and is typically located at unmethylated promoters of lowly transcribed genes. In embryonic stem cells, bivalent chromatin has been proposed to poise developmental genes for future activation, silencing or stable repression upon lineage commitment. Normally, bivalent chromatin is kept in tight balance in cells, in part through the activity of the MLL/COMPASS-like and Polycomb repressive complexes that deposit the H3K4me3 and H3K27me3 modifications, respectively, but also emerging novel regulators including DPPA2/4, QSER1, BEND3, TET1 and METTL14. In cancers, both the deregulation of existing domains and the creation of *de novo* bivalent states is associated with either the activation or silencing of transcriptional programmes. This may facilitate diverse aspects of cancer pathology including epithelial-to-mesenchymal plasticity, chemoresistance and immune evasion. Here, we review current methods for detecting bivalent chromatin and discuss the factors involved in the formation and fine-tuning of bivalent domains. Finally, we examine how the deregulation of chromatin bivalency in the context of cancer could facilitate and/or reflect cancer cell adaptation. We propose a model in which bivalent chromatin represents a dynamic balance between otherwise opposing states, where the underlying DNA sequence is primed for the future activation or repression. Shifting this balance in any direction disrupts the tight equilibrium and tips cells into an altered epigenetic and phenotypic space, facilitating both developmental and cancer processes.

## Introduction

Cancer can be described as a disease of cellular identity and is often associated with a dedifferentiated state recapitulating epigenetic, transcriptomic and phenotypic properties associated with embryonic cells [1–3]. Emerging models propose that cancer cells can hijack developmental programmes to increase their plasticity and facilitate their evolution and adaptation [4–7]. In both cancer and development, cellular identity is controlled in part by epigenetic mechanisms, involving DNA- and chromatin-modifying complexes. These epigenetic mechanisms collaborate in a co-ordinated manner to orchestrate gene expression changes associated with differentiation and are often compromised during cancer initiation and progression [8].

**Figure 1.**
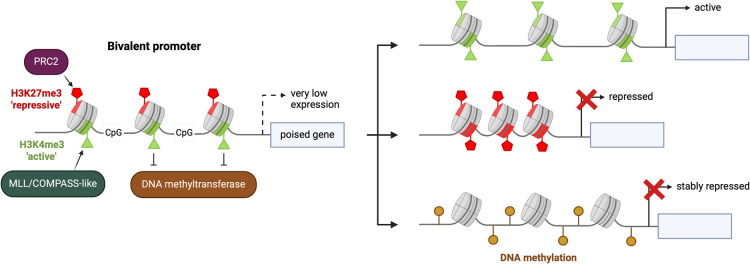
Developmental resolution paths for bivalent chromatin. Bivalent promoters are defined by the co-occurrence of active-associated H3K4me3 (green triangles) and inactive-associated H3K27me3 modifications (red pentagons) on opposing tails of the same nucleosome (grey pucks). These marks are deposited by the MLL/COMPASS-like and PRC2 complexes, respectively. The poised lowly expressed state of bivalent promoters can be resolved to an active H3K4me3-only (top), inactive H3K27me3-only (middle) or stably repressed DNA methylated (bottom) state.

Nucleosomes, the fundamental unit of chromatin, are comprised of histone proteins and associated DNA. Changes in histone tail modifications influence chromatin structure controlling the availability of DNA for networks of sequence-specific transcription factors [9–11]. In this review, we focus on the bivalent chromatin structure which is defined by the co-occurrence of trimethylation of lysine 27 on histone-H3 (H3K27me3) and trimethylation of lysine 4 on histone-H3 (H3K4me3) on opposing tails within the same nucleosome [12,13] (Figure 1). Generally, these two marks are associated with gene repression and activation respectively. However, their co-occurrence at lowly transcribed promoters occupied by paused RNA Polymerase II has been documented across a wide variety of species and cellular contexts ranging from fungi, plants and zebrafish to mouse and human embryonic and pluripotent cells [14–26]. The existence of bivalent chromatin across different species suggests the functional importance of bivalent domains may be conserved.

Bivalent chromatin is typically found at CpG island containing promoters. Throughout mammalian genomes, ∼70–80% of CG dinucleotides are methylated; however, CpG islands are typically devoid of DNA methylation [27]. The first genome-wide maps of bivalent chromatin in mouse embryonic stem cells (ESCs) revealed that almost all CpG-rich promoters were marked by H3K4me3 and a subset (∼20%) of these were also marked by H3K27me3 [28,29]. Current models propose that bivalent chromatin plays a role in pluripotency by poising differentiation and lineage commitment genes for future activation or repression [28–30], although it should be noted that causality has not been demonstrated. During *in vitro* differentiation of mouse ESCs, bivalent domains were shown to resolve to either a H3K4me3 highly expressed, or H3K27me3 silenced state [28,29] (Figure 1). Alternatively, loss of both H3K4me3 and H3K27me3 at bivalent regions could result in a stably repressed state marked by DNA methylation (Figure 1).

While traditionally studied in the context of pluripotency, emerging studies point to a role for bivalent chromatin in facilitating cellular adaptation in a range of other contexts [16,17,23]. Changes in bivalent chromatin at key cancer-related genes have been associated with cancer initiation and adaptation to therapy [31,32]. Several recent reviews have detailed the regulation of bivalent chromatin in stem cells and during development [33,34], here we shed light on recent advances in our understanding of how this chromatin signature is redeployed in cancers. First, we will discuss approaches used to measure bivalent chromatin, followed by a discussion on the enzymatic complexes required to form H3K4me3 and H3K27me3, and how they may be targeted specifically to form bivalent chromatin. Lastly, we will discuss how bivalency could reflect distinct regulatory mechanisms for cancer cells to maintain the epigenetic plasticity necessary for adaptation.

## Techniques to accurately detect and measure bivalent chromatin

Most observations of bivalent chromatin have come from chromatin immunoprecipitation (ChIP)-based approaches [35] to profile H3K4me3 and H3K27me3 at loci of interest (by qPCR) or genome-wide (by high throughput sequencing). Alternatively, CUT&RUN and CUT&Tag methods [36] can be similarly used to profile H3K4me3 and H3K27me3. These approaches typically profile a single modification at a time and so bivalent chromatin is inferred bioinformatically as regions independently enriched for both H3K4me3 and H3K27me3. However, this approach does not control for allelic or cellular heterogeneity in the sample where the two modifications exist on different alleles or in different cells. Consequently, high false-positive rates ranging from 14% in human T cells [37] to ∼25% in mouse ESCs [38,39] have been reported using this *in silico* overlap approach. Thus, while this approach may provide preliminary insights, further confirmation of true bivalency is required. To conclusively demonstrate true bivalent chromatin where both modifications occur on the same nucleosome, sequential ChIP or ChIP–reChIP methods can be used [37–39]. This involves two successive ChIP reactions, whereby the first purification of either H3K4me3- or H3K27me3-containing nucleosomes is then subjected to a second immunoprecipitation for the alternative modification. In more heterogenous populations such as cancer samples, sequential ChIP can rule out sample or allelic heterogeneity and consequently has increased accuracy. Moreover, the higher sensitivity can enable detection of bivalent chromatin in a subset of cells [38]. Sequential ChIP is recommended to confirm bivalent loci and/or profile cell types and conditions where the bivalent landscape is unknown. One limitation is that sequential ChIP typically requires high amounts of starting material, although recently a protocol for profiling 2 million cells has been reported [38]. To profile smaller populations, or even single cells, CUT&Tag-based methods may be more appropriate. Recently, CUT&Tag was adapted to use barcoded-labelled antibodies to simultaneously map multiple histone modifications in single cells [40]. While it has not yet been shown for H3K4me3–H3K27me3 bivalent regions, this approach could be useful, especially in complex or rare populations.

Quantitative genomic approaches are becoming increasingly necessary for accurate dissection of chromatin dynamics and regulation. An important consideration and drawback of sequential ChIP is that it does not provide absolute levels of bivalent chromatin. While spike-in controls are used in single ChIP-seq to help normalisation between samples and conditions [41–43] they have not yet been adopted for sequential ChIP. Alternatively, mass spectrometry can provide the absolute abundance of histone modifications at a population level, albeit at the expense of locus-specific information [13].

Complementing genomic approaches, imaging-based methods can visualise bivalent chromatin in cells, trading locus information for single-cell and sub-cellular resolution. In ESCs, iChmo, an *in situ* proximity ligation assay-based approach, was used to profile bivalent chromatin dynamics in heterogeneous ESCs and upon differentiation [44]. Another approach involving engineered probes containing H3K27me3 and H3K4me3 reader domains fused to fluorescent reporters allows visualisation of bivalent chromatin in living cells [30,45,46], and coupling these reporters with biotin can facilitate genomic mapping via streptavidin pull down [46]. These approaches come with the caveat that reader domains bound to just one or neither histone modification will also fluoresce and so relies on signal enrichment within the nucleus.

In summary, multiple approaches are available to study bivalent chromatin, each with their advantages and disadvantages. A major challenge facing the field is that most studies define bivalent chromatin *in silico* from independently generated ChIP-seq or similar datasets. Thus, care must be taken when extrapolating findings from these studies as they may be confounded by high false-positive and false-negative rates. Here, we review studies defining bivalent chromatin using all approaches mentioned here, noting when true bivalency has been comprehensively shown.

## Formation of bivalent chromatin domains

A lot is now known about the histone lysine methyltransferases that catalyse trimethylation of H3K4 and H3K27. In mammals, Polycomb repressive complex 2 (PRC2) deposits H3K27me3 while SET1/COMPASS or MLL/COMPASS-like group protein complexes deposit H3K4me3. The molecular details of these complexes and their roles in development and transcription have been comprehensively reviewed [9,47,48]. Here, we provide insights into how these complexes interact with and shape bivalent domains.

### Formation of bivalent chromatin domains — H3K4me3

Methylation of lysine 4 on histone H3 (H3K4) is one of the most extensively studied histone modifications due to its links with transcription and mis-regulation in cancer [47]. In mammals H3K4 methylation is deposited by SET1/COMPASS and MLL/COMPASS-like complexes that contain shared core subunits and one of six lysine methyltransferases: SET1A (KMT2F), SET1B (KMT2G), MLL1 (KMT2A), MLL2 (KMT2B), MLL3 (KMT2C) and MLL4 (KMT2D). H3K4me1 is catalysed by MLL3/4 and is associated with enhancer elements, while promoter-enriched H3K4me2/3 is deposited by SET1A/B and MLL1/2 [49,50]. Targeting of SET1/COMPASS and MLL/COMPASS-like complexes to non-methylated CpG islands is at least partly mediated through DNA binding zinc finger CxxC domains contained within the histone-methyltransferases (HMT) themselves or associated subunits [51–53]. SET1A/B complexes are enriched at actively transcribed genes, while MLL1/2-containing complexes are broadly distributed across the genome [54,55].

**Figure 2.**
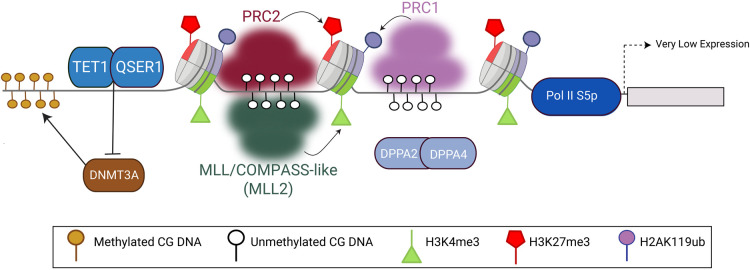
Molecular players at bivalent domains. Schematic of a bivalent domain containing H3K4me3 (green triangles), H3K27me3 (red pentagons) and H2AK119Ub (purple circles) at a lowly transcribed gene promoter containing paused RNA Polymerase II. Bivalent domains are typically devoid of DNA methylation (white circles). Molecular complexes involved are highlighted in the same colour as the mark they catalyse. Other molecular players implicated in regulating bivalent chromatin are shown along with arrows linking them with their proposed modes of action when known.

Of the six lysine methyltransferases, MLL2 has been implicated to be most important at bivalent sites, at least in ESCs [54,55]. Consistent with their low levels of transcription, bivalent promoters are enriched for MLL2-containing complexes and devoid of SET1A/B complexes (Figure 2) [56]. Depletion of *Mll2* but not *Mll1* causes a reduction in H3K4me3 at bivalent promoters [39,54,56], supporting a primary role for MLL2 at bivalent promoters in pluripotent cells [54,55]. This lack of redundancy between MLL1 and MLL2-containing complexes at bivalent promoters in pluripotent cells is surprising given the highly similar subcomplex assemblies [54,55]. In contrast with ESCs, both MLL1 and MLL2 have been implicated in the deposition of H3K4me3 at bivalent genes in a cell culture model of chronic myeloid leukaemia (CML) [57]. This highlights the need to independently consider the roles of MLL1 and MLL2 at bivalent domains in different cellular contexts.

MLL1/2 containing complexes specifically contain the scaffold protein Menin [58], which binds to the N-terminus of MLL1/2 in normal cells as well as oncogenic MLL1-fusion protein complexes [58,59]. While the exact role of Menin in these complexes remains unclear, Menin may act by facilitating transcriptional activation by mediating the recruitment of MLL1/2 to chromatin [60]. In the context of bivalent chromatin, the role of Menin is even more ambiguous. In the same model of CML as above, loss of Menin caused a redistribution of MLL1 away from highly transcribed genes to bivalent genes and was associated with an increase in H3K4me3 and transcription of the bivalent genes [57]. This suggests, in the context of this CML model, Menin restricts the expression of bivalent genes. Menin also binds oncogenic MLL1-fusion protein complexes [58,59], and so it will be interesting to explore its role in these cancer contexts. Taken together these data suggest that the role of MLL1/2 and Menin at bivalent domains is likely cell-type and context dependent.

### Formation of bivalent chromatin domains — H3K27me3

The repressive component of bivalent chromatin, H3K27me3, is deposited by PRC2 [9]. PRC2 is essential for regulating cellular identity during differentiation and development in a wide range of species [48], and mutations in PRC2 are associated with developmental disorders and malignancies including lymphomas and gliomas [61–63]. H3K27me3 is deposited at low levels throughout the genome, and is highly enriched at promoters of developmental genes [64,65], which typically display a bivalent signature [25,28,66–68]. The core subunits of PRC2 are comprised of EED, SUZ12 and the HMT EZH1 or EZH2, in association with RBBP4/7 (Figure 2). These core PRC2 subunits associate with several accessory proteins which define two main subassemblies, PRC2.1 and PRC2.2 [69,70], which fine-tune the recruitment of PRC2 to target genes [65,71,72]. The mechanisms of PRC2 recruitment to target genes have been reviewed in detail elsewhere [73–78]. Further studies are needed to investigate the role of different assemblies of Polycomb complexes in the formation and regulation of bivalent chromatin domains in different cellular contexts. PRC2.1 is the dominant subcomplex in terms of H3K27me3 deposition [65,71,72,79] which has led some studies to specifically implicate it in relation to bivalency [57,80]. However, since PRC2.1 and PRC2.2 co-localise at the vast majority of target genes in mouse pluripotent cells [65,71,72], it will be important to also consider the role of PRC2.2 at bivalent domains.

In addition to PRC2, the Polycomb system also includes Polycomb repressive complex-1 (PRC1). PRC1 comes in both canonical (cPRC1) and variant (vPRC1) forms and catalyses mono-ubiquitination at lysine 119 of histone H2A (H2AK119ub) [9] (Figure 2). The plethora of mechanistic studies in pluripotent cells in recent years has highlighted the intricate cross-talk between Polycomb subassemblies required to maintain genes in a repressive state. Loss of H2AK119ub leads to a reduction in PRC2 binding and a rapid induction of Polycomb target gene expression, including bivalent genes. This underscores H2AK119ub as a key gatekeeper of Polycomb-mediated gene repression both broadly but also at bivalent chromatin [81–83]. Together, these studies highlight the likely role for PRC1 and H2AK119ub in establishing bivalent domains and maintaining them in a low expression state [34]. Therefore, as we move past studies of bivalency in pluripotent cells and think about this chromatin structure in the context of cancer initiation and progression we must consider other marks, such as H2AK119ub, and the variety of complex assemblies that intersect at these domains.

### Formation of bivalent chromatin domains — other players

Recent studies in mouse ESCs have uncovered additional players in maintaining bivalent domains that act through antagonising DNA methylation or regulating H3K27me3 levels [84–88]. DNA at bivalent regions is generally unmethylated, and so bivalent chromatin has been proposed as a mechanism to protect CpG islands from aberrant DNA methylation [89]. The ten–eleven translocation (TET) enzymes are involved in the active turnover of DNA methylation in a variety of biological processes [90]. Loss of all three TET enzymes (TET1–3) in either mouse or human ESCs induces focal DNA hypermethylation most notably at bivalent promoters, in addition to other gene regulatory regions [91,92]. Taken together, these data support a role for TET enzymes in safeguarding bivalent promoters from aberrant DNA methylation. Similarly, glutamine serine rich protein 1 (QSER1) has emerged as a novel protein protecting developmental-associated bivalent domains from *de novo* DNA methylation in human ESCs [84]. Mechanistically, QSER1 localises to regions flanking bivalent domains, associates with TET1 and antagonises DNMT3A. Consequently, loss of QSER1 induces DNMT3A-mediated DNA hypermethylation at bivalent genes. Taken together, these data support a role for TETs and TET-associated proteins in protecting bivalent promoters from aberrant DNA methylation.

Developmental pluripotency associated 2 and 4 (DPPA2/4) proteins have recently been discovered as new regulators of bivalent chromatin in mouse ESCs [85,86]. Single ChIP [85,86] and reChIP [38] analyses have revealed loss of DPPA2/4 causes focal reduction in both H3K4me3 and H3K27me3 at some, but not all, bivalent promoters. Loss of bivalency is associated with an accumulation of DNA methylation. DPPA2/4 are non-enzymatic proteins that interact strongly with chromatin, even under high salt concentrations [93], and interact with Polycomb and MLL/COMPASS-like complexes amongst other chromatin factors [85,93]. This suggests that at bivalent regions they may act as molecular anchors to stabilise or fine-tune the interactions for other regulatory complexes, such as Polycomb or MLL/COMPASS-like complexes at bivalent chromatin.

BEN domain containing 3 (BEND3) and methyltransferase 14 (METTL14) have also recently been implicated in regulating levels of H3K27me3 at bivalent promoters [87,88,94,95]. Loss of either protein causes a marked decrease in PRC2 binding and H3K27me3 levels at bivalent promoters [87,88]. While the mechanistic details of how this occurs remain to be seen, we know that the loss of BEND3 also leads to a global increase in DNA methylation [95] suggesting it has wide-reaching influences beyond bivalent domains. The role of METTL14 at bivalent domains is independent of its more studied function with METTL3 in modifying RNA [96,97]. It is tempting to speculate that METTL14 somehow regulates the equilibrium between H3K4 and H3K27 modifying complexes since the reduced H3K27me3 simultaneously leads to an increase in H3K4me3 and gene transcription [87]. More functional studies uncoupling the METTL3-dependent and -independent roles of METTL14 could provide more mechanistic insights.

These studies illustrate that the regulation and fine-tuning of the bivalent chromatin environment extends beyond the catalytic complexes to include a range of additional molecular factors, including, but not necessarily limited to, those described above. It is now critical we expand on these studies to identify other regulators, understand their functional and mechanistic modes of action, while also considering their roles in regulating bivalent domains in other contexts, including cancer.

## Bivalent chromatin and cancer

It is now clear that, in addition to genetic changes, disruption of the chromatin and transcriptional environments is a major driving mechanism in cancer [98]. There is growing evidence that cancers can reawaken embryonic programmes to facilitate many aspects of tumorigenesis including drug resistance, metastasis and immune evasion [5,7,99]. Like embryonic cells, cancers can also have heightened plasticity [99] and it is tempting to speculate that aspects of epigenetic plasticity found in embryonic cells, such as bivalent chromatin, may similarly play a role in facilitating cancer cell adaptation.

**Figure 3.**
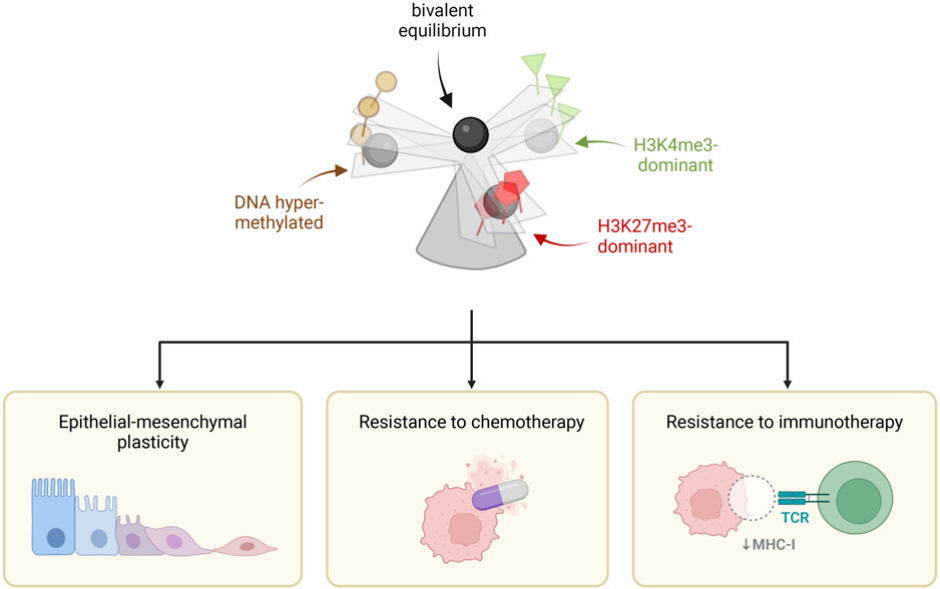
Disrupting the balance of bivalent chromatin in cancer. Bivalent chromatin exists in fine equilibrium (top panel) and tipping the balance in any direction alters the chromatin state and ultimately influences transcriptional programmes in the cell. This may affect many aspects of cancer evolution including epithelial-to-mesenchymal plasticity (bottom left), drug resistance (bottom centre) and immunotherapy resistance (bottom right). H3K4me3 is depicted by green triangle, H3K27me3 by red pentagon and DNA methylation by brown circle.

Changes to the bivalent chromatin landscape have been specifically implicated in different aspects of multiple cancers, including glioma [100], neuroblastoma [101], breast [102], colon [31] and melanoma [103]. It is important to note that most of these studies have inferred bivalency from single ChIP datasets, and there are limited comprehensive sequential ChIP maps of the dynamics of bivalent chromatin in the context of cancer. In this section, we review how disrupting the epigenetic balance at bivalent domains may promote cancer processes including epithelial-to-mesenchymal transition (EMT), chemotherapy resistance and immune evasion (Figure 3).

### Bivalent chromatin and epithelial to mesenchymal transition

EMT is a cellular process whereby immobile epithelial cells shift to a migratory mesenchymal phenotype. While the EMT programme is a normal part of embryogenesis, it also contributes to tissue regeneration and promotes cancer metastasis [104,105]. A network of core EMT-inducing transcription factors including Slug, Snail, Twist and Zeb1 are required to implement EMT [106]. Importantly, this is not a simple binary switch but a reversible progression through intermediate states, wherein cells can co-express both epithelial and mesenchymal characteristics [107,108], implicating epigenetic regulation as a key component of these transitions.

Bivalent domains have been shown to regulate EMT transcription factor genes in breast cancer [102]. Chaffer and colleagues revealed that the chromatin configuration within breast cancer cells lines has an important role in the cell's ability to activate the EMT programme in response to transforming growth factor beta (TGF-β). In the more aggressive basal subtype of breast cancer, cells maintain the *Zeb1* promoter in a bivalent configuration, facilitating EMT induction. In contrast, in luminal-type breast cancer cells, which are unresponsive to EMT-inducing signals, the *Zeb1* promoter is marked only by H3K27me3, locking the promoter in a repressed state [102].

Similarly, profiling of 46 melanoma samples including melanoma samples, patient-derived cultures and cell lines revealed that a differential putative bivalent chromatin state separated the *NRAS* and *BRAF* mutant melanoma subtypes [103]. Using Roadmap data from primary melanocytes, and *BRAF* and *NRAS* mutant melanocytes, the authors noted that as melanocytes progressed towards melanoma, *BRAF* mutants exhibited a global decrease in the number of bivalent domains, while *NRAS* mutants had an increase. This increase is likely a consequence of increased expression of PRC2 subunit SUZ12 [103]. It is interesting to note that despite the global increase in bivalent domains, site-specific gains and losses at functionally relevant genes drive phenotypic changes. For example, at EMT-associated genes, the resolution of bivalency in melanocytes to a broad H3K4me3-only state in cancer cells could enable transcription at these sites and help drive metastasis. In contrast, the gain of tumour-specific bivalent domains at genes involved in apical junctions may contribute to a more mesenchymal phenotype [103].

These two studies highlight how the balance of bivalent chromatin can dictate how cancer cells respond to external signals and undergo EMT. Supporting this, a recent screen for EMT regulators highlighted PRC2 and MLL4 (KMT2D) as machinery governing epithelial-to-mesenchymal plasticity [109]. Interestingly, the loss of either complex directed cells toward distinct EMT states along the EMT spectrum [109], highlighting the need for continuous balance among chromatin-modifying complexes.

### Bivalent chromatin and resistance to immunotherapy

Immunotherapy has revolutionised cancer treatment by promoting cytotoxic T cells to recognise and kill cancer cells displaying antigens bound to major histocompatibility complex class I (MHC-I) molecules. Unfortunately, many cancers are either unable to be targeted with immunotherapy or develop resistance by silencing MHC-I expression. In embryonic and pluripotent cells, the MHC-I genes are bivalently marked, and this is normally resolved to an active H3K4me3 state in adult somatic tissues [57,110]. In contrast, cancer cells can silence MHC-I expression, either through genetic mutations [111] or by regaining the pluripotency-associated bivalent signature [110]. The presence of PRC2 at these loci restricts transcriptional induction of MHC-I in response to cytokine stimulation, thus driving resistance to T cell-mediated killing [110]. While the exact mechanism remains unclear, recent work has suggested the MLL/COMPASS-like complex member Menin can also facilitate repression at this locus [57]. Importantly, immunotherapy resistance can be disrupted by treatment with either PRC2 and/or Menin inhibitors [57,110]. In an analogous situation, recent sequential ChIP analyses in *Fusarium graminearum* suggest the fungal pathogen uses bivalent chromatin to dampen the expression of virulence genes following infection to avoid host recognition [16]. While more work is required to elucidate the precise mechanisms involved, these studies suggest that tipping the balance of bivalent chromatin may render cancer cells more susceptible to treatments such as immunotherapy and potentially represent novel therapeutic avenues.

### Bivalent chromatin and resistance to chemotherapy

Resistance to chemotherapy is another major challenge in successfully treating cancer patients. Drug-induced cellular reprogramming of the transcriptional and chromatin landscape has emerged as a non-genetic mechanism of chemotherapy resistance in multiple cancer types [112–114]. In some models, following chemotherapy treatment, a population of transcriptionally distinct drug-tolerant persister cells remains, and it is thought these cells may contribute to recurrence. A recent study [32] modelling chemoresistance in triple-negative breast cancer detected bivalent domains at a subset of genes within the persister cell transcriptional programme, confirming these regions as truly bivalent by sequential ChIP [32]. Treating these cell lines and patient samples with chemotherapy led to a loss of H3K27me3 at a subset of the bivalent regions, inducing the persister cell transcriptional programme. If H3K27me3 levels were sustained by simultaneous co-treatment with an H3K27 demethylase inhibitor (KDM6i), the number of persister cells decreased [32]. However, it is important note that treatment with the KDM6i is not specific to bivalent domains and would sustain H3K27me3 globally. These data suggest that bivalent chromatin may poise a persister cell transcriptional programme in untreated cancer cells, facilitating therapy resistance, and highlights how the timing of epigenetic therapies is essential in achieving the desired outcomes.

Taken together these studies highlight the ability of cancer cells to manipulate the plasticity associated with bivalent promoters. Tweaking the chromatin landscape through either loss or gain of H3K4me3 or H3K27me3, around key genes involved in metastases, immune evasion and drug resistance, can be sufficient to promote cancer phenotypes (Figure 3). Therefore, more detailed insights into the temporal changes in bivalent signatures during cancer progression and following drug treatment could help inform better treatment options.

## Bivalent chromatin: striking a balance

We propose that bivalent chromatin is not static but dynamic and must be actively maintained or faithfully re-established at its target loci [115] (Figure 3). Supporting this, mathematical modelling predicts bivalent chromatin is bistable and switching rapidly between active and silent states [116]. It has been proposed that this bistable state is upheld by the underlying CpG islands at gene promoters which can be continuously sampled by H3K4 and H3K27 methyltransferases [9]. Like a seesaw, the otherwise opposing H3K4me3 and H3K27me3 are kept in tight equilibrium at DNA hypomethylated loci [117,118]. Failure to actively maintain one modification could tip the balance and lead to a complete dominance of another state. Thus, bivalent chromatin reflects this bistability as fine dynamic balance between competing marks, marking domains poised for any number of end-states.

Bivalent chromatin and DNA methylation are generally mutually exclusive. Several lines of evidence suggest that promoters marked by bivalent chromatin in ESCs become deregulated in cancers [119,120]. Indeed, analysis of DNA methylation data from patient samples in The Cancer Genome Atlas revealed that 75% of genes hypermethylated in cancer were bivalently marked in human ESCs [121]. The quest for continuous equilibrium between DNA methylation, H3K4me3 and H3K27me3 at these sites is evident by the restoration of bivalency following chemically induced genome-wide DNA demethylation [120,122].

In both cancer cells and ESCs, tipping the bivalent chromatin balance in any direction would lead to altered transcriptional programmes and altered cellular identity and/or functions. In development, the resolution of bivalent chromatin to an H3K4me3-only state enables lineage-specific transcription factor expression and lineage commitment. Similarly, in cancer cells, resolution to an H3K4me3-only state may activate transcriptional programmes such as EMT or chemoresistance if the necessary transcription factors are available. Conversely, re-establishing bivalent chromatin at active loci in cancer cells may dampen transcription, such as MHC-I genes, and diminish gene expression programmes that are otherwise detrimental to the cancer cell. Comprehensive genome-wide maps of *bone-fide* bivalent chromatin during cancer cell adaptation and evolution are needed to tease apart both the resolution and *de novo* formation of bivalent chromatin, and the dynamic interplay and balance between bivalent chromatin, and the variety of trans-acting mechanisms involved in establishing gene expression programmes associated with cancer cell behaviours.

This balancing act does not occur in isolation and is impacted by other regulators and layers of epigenetic information. Factors involved in the regulation of developmental bivalent chromatin are often deregulated cancer. For example, *Dppa2/4* and *Qser1* are up-regulated in multiple cancer types, and this is often associated with poor prognosis [4,123–126], and mutations or deregulation of TET proteins drive a wide range of malignancies [127,128]. Moreover, subunits of the Polycomb and MLL/COMPASS-like complexes are deregulated in a range of cancers [63,129]; however, how this alters bivalent chromatin has not been investigated. While bivalent chromatin associates with poised regulatory elements, causality has not been shown and this structure may simply represent competing chromatin-regulatory processes at these sites. We are not aware of any ‘readers’ of bivalent chromatin in mammalian systems, although these have been discovered in plants [130]. Moreover, while bivalent chromatin is generally devoid of DNA methylation, how this seemingly incompatible layer of chromatin-based information shapes and is shaped by the bivalent chromatin landscape remains to be disentangled mechanistically. Going forward it will be important to explore not only the dynamics and implications of bivalent chromatin in cancer but also understand how the mis-regulation of regulatory complexes at bivalent domains contributes to cancer development and may represent novel therapeutic vulnerabilities.

## Perspectives

- Bivalent chromatin is an exemplar of epigenetic plasticity in developmental and cancer contexts. Co-occurrence of H3K4me3 and H3K27me3 at poised gene promoters is thought to facilitate future changes in transcriptional programmes and activation into an H3K4me3-only state, repression by H3K27me3 or stable silencing by DNA methylation.
- Bivalent chromatin represents a fine balance between active H3K4me3 and repressive H3K27me3 modifications underpinned by DNA hypomethylation. Tipping the balance in any direction disrupts this epigenetic equilibrium, shifting cells into an altered epigenetic and phenotypic space.
- Future genome-wide maps of *bone-fide* bivalent chromatin dynamics along with the discovery of novel regulators will enable an understanding of how this structure is established and resolved during development and cancer evolution. Together these insights will strengthen or even revise our understanding of bivalency in the great scheme of cellular decisions that underlie diverse cellular trajectories.

## Open Access

Open access for this article was enabled by the participation of University of Melbourne in an all-inclusive *Read & Publish* agreement with Portland Press and the Biochemical Society under a transformative agreement with CAUL.

## Abbreviations

ChIP: chromatin immunoprecipitation
CML: chronic myeloid leukaemia
EMT: epithelial-to-mesenchymal transition
ESCs: embryonic stem cells
HMT: histone-methyltransferases
MHC-I: major histocompatibility complex class I
TET: ten–eleven translocation
